# Body size dynamics in young adults: 8-year follow up of cohorts in Brazil and Thailand

**DOI:** 10.1038/nutd.2016.24

**Published:** 2016-07-18

**Authors:** V Yiengprugsawan, B L Horta, J V S Motta, D Gigante, S-A Seubsman, A Sleigh

**Affiliations:** 1 Centre for Research on Ageing, Health & Wellbeing and National Centre for Epidemiology and Population Health, Research School of Population Health, The Australian National University, Canberra, Australian Capital Territory, Australia; 2 Postgraduate Programme in Epidemiology, Universidade Federal de Pelotas, Pelotas, Brazil; 3 Postgraduate Programme in Health and Behavior, Universidade Católica de Pelotas, Pelotas, Brazil; 4 School of Human Ecology, Sukhothai Thammathirat Open University, Nonthaburi, Thailand

## Abstract

Increase in body size has appeared as an epidemic in Western countries and is now rapidly emerging in low- and middle-income countries, contributing to the rise in non-communicable diseases worldwide. Brazil and Thailand have gone through similar economic and health transitions, and this unique comparative study investigates changes in body size (body mass index) in relation to socioeconomic status in two cohorts of similar age followed from 2004/2005 to 2012/2013. At 20–24 years of age, Pelotas cohort members had a much higher prevalence of overweight and obesity (20.7 and 8.6%) than the Thai cohort (6.0 and 1.7%); these proportions rose to 34.6% and 22.9% vs 15.8% and 5.1%, respectively, in their early 30s. An association between a higher socioeconomic status and increase in overweight and obesity was observed among males; but an inverse pattern was noted for females in both cohorts and remained statistically significant after 8 years of follow up. Our comparative longitudinal analyses highlight the relationship between two middle-income settings facing rapid increases in body size (2–3 fold increase in the rate of overweight and obesity). Long-term follow up and a lifecourse approach for effective prevention of obesity will minimize adverse health burdens in later life.

## Introduction

The prevalence of overweight and obesity has substantially increased in high- and middle-income countries over the last two decades. Indeed, obesity is now a major determinant of the global burden of chronic non-communicable diseases, especially of metabolic and cardiovascular diseases.^1, 2^ Weight gain, long a feature of middle age and older adults, now is occurring much earlier in the life span. This reflects increasing exposure to high-calorie diets and physical inactivity.^3, 4^

In Western countries, higher socioeconomic groups—especially females—are less likely to be obese, but evidence is limited regarding such an association in developing economies in non-Western settings.^5, 6^ In middle-income countries, the inverse relationship of obesity and socioeconomic status (SES) has already been noted among females but it is infrequently reported for males.^5, 7, 8^

Brazil and Thailand are middle-income countries with different cultures and social geography, however they have faced similar economic and social transitions since industrializing in the second half of the twentieth century. Both countries have successfully implemented primary health-care systems and the majority of their citizens have free access to universal health care.^9^ In the past few decades, both countries have also undergone rapid epidemiological transitions from widespread infectious diseases, mother–child mortality and child malnutrition to lower mortality and emerging chronic diseases.^10^

In this study, we purposely selected two countries representing similar positions in socioeconomic and health transition trajectories. In particular, we will investigate one of the prominent risk factors in metabolic and cardiovascular diseases—changing body size and increasing obesity among young to middle-aged Thai and Brazilian adults—using two prospective longitudinal data sets spanning the past decade. We hypothesized that despite differences in culture, diets and body composition, and environments, results could shed light in divergent or convergent pathways on weight changes and relationships with SES in these two settings. Findings will add to limited cross-country evidence in monitoring global public health challenges such as the obesity epidemic and provide insights into rapidly transitioning populations in middle-income economies.

## Subjects and methods

### Study design

We compared data from two cohort studies—the 1982 Pelotas Birth Cohort (one of the longest-running birth cohorts in developing countries) and the Thai Cohort Study, which has been running for the last 10 years successfully following adult open university students living off-campus nationwide.^11, 12^ Both cohorts were followed up 8 years later—in 2012–2013 (Pelotas) and in 2013 (Thai). At that point, about one-fifth of the Thai cohort members were of similar age to the Pelotas cohort.

The 1982 Pelotas Birth Cohort Study included at baseline 5914 live births whose families lived in Pelotas, a Southern Brazilian city. This study population has been followed up several times, initially focused on perinatal, infant, and early childhood morbidity and mortality.^11^ In 2012–2013, 3701 cohort members were interviewed (68.1% follow-up rate). At this time, Pelotas cohort members were entering adulthood and risk factors for chronic disease were evaluated (2013), including smoking, diet, physical activity and body mass index (BMI).^13^

The Thai Cohort Study continues to document and analyze transitional patterns of health risks and outcomes. The original research cohort included 87 151 distance-learning adult students enrolled at Sukhothai Thammathirat Open University who completed the baseline mail-out questionnaire in 2005. Their mean age was 29 years, slightly more than half were females, and half resided in urban areas.^12, 14^ The cohort was subsequently followed up in 2009 and 2013 (>70% at each wave); the 2013 group numbered about 43 000 and those aged 19–25 years at 2005 at baseline (*n*=9893) are reported here. This Thai group matches the Pelotas cohort for birth years so that the comparative analyses cannot be confounded by age.

### Exposures and outcomes:

- BMI was derived from weight and height noted at both 2004/2005 and 2012/2013 examinations. In the Pelotas cohort, Western BMI category cutoffs were applied (BMI<18.5 as ‘underweight', 18.5–<25 as ‘normal', 25–<30 as ‘overweight', and 30+ as ‘obese'). For the Thai cohort, weight and height were used to create BMI by both Western and Asian cutoffs; the latter follow the International Obesity Task Force guidelines with BMI 18.5–<23 as ‘normal', 23–<25 as ‘overweight at risk', and 25+ as ‘overweight and obese'.^15^
- Variables included SES derived from personal monthly income. In the Pelotas cohort data in 2004/2005, the Brazilian Real income was divided into thirds. In the Thai cohort, personal monthly income in Baht were reported in three ordinal categories: <3000 Baht was the lowest (20.2%); 3001–7000 Thai Baht (47.5%); and >7000 Baht (32.3%). Sex, education and health behaviors (smoking, regular alcohol drinking and physical activity) were noted in both cohorts.

## Results and discussion

Both cohorts were aged about 20–24 years at the baseline and the proportion of males was 51.3% in the Pelotas cohort and 32.8% in the Thai cohort (Table 1). A notable difference was that the Pelotas cohort members predominantly resided in urban areas compared with 43.9% of the Thai cohort. Also, all Thai cohort members had completed at least 9 years of education at baseline compared with 69.9% of the Pelotas cohort members.

Based on BMI in both cohorts aged 20–24 years, Pelotas cohort members at baseline had a substantially higher prevalence of overweight or obesity (20.6 and 8.3%) than the Thai cohort members (5.3 and 1.6%). Most Thai cohort members had a much smaller body size and the prevalence of underweight was higher (25.3% compared with 4.4% in the Pelotas cohort) and especially among Thai females (31.7% compared with 5.2% in the Pelotas cohort). With respect to health behaviors, smoking and alcohol drinking were reported at very low rates among Thai females, partly due to gender-specific cultural differences. In both cohorts, a higher proportion of males reported weekly physical activity of >150  min.

In Table 2, both cohorts were categorized by SES and BMI patterns in both 2004/2005 baseline and 8 years later. In the Pelotas cohort, the increase in proportion in overweight and obesity is mostly attributed to baseline cohort members who initially had normal BMI. However, in the Thai study, there was a much higher proportion of cohort members at baseline, hence, an increase in weight shifted from underweight to normal, as well as normal to overweight and obesity. Varying patterns observed could be due to differences in body compositions of the two cohorts throughout childhood and adolescence. Among the Pelotas cohort members at the 8-year follow up, the relationship of increasing body size was associated with higher SES among males and lower SES among females. In the Thai cohort, a positive SES male relationship persisted for overweight and obesity; in contrast, the inverse relationship between rising income and lower BMI among females was most notable with the Asian cutoff but the effect was attenuated over time.

Current literature for low- and middle-income countries on the gender–SES–obesity relationship is subject to the inherent complexity of different SES and obesity measures.^5, 7^ Overall available evidence generally supports our findings that overweight and obesity tends to increase with SES for males and to decrease for females. For example, a study of adult obesity in Brazil reported that education was not associated with obesity in men but women with higher education had lower obesity rates.^16^ Parallel findings were also observed among young- and middle-aged Peruvians.^17^ In middle-income Asia, a Chinese study reported that SES was positively associated with overweight and obesity among males, whereas high–status females were less likely to be overweight or obese.^18^ Another study among Malays in Singapore revealed that prevalence of overweight and obesity decreased with SES for females but increased for males based on different SES measures.^19^ Similar SES obesity findings were also reported from the Thai National Health Examination Survey; men with higher education and female with lower education tended to be obese.^20^

The main strength of our study is the comparison between two cohorts in the same age range with key comparable outcomes during the same period of 8-year follow up. Some differences are notable due to the composition of the two cohorts; the Pelotas cohort was generated by birth in one city (urban areas) and the Thai cohort includes nationwide (half urban and half rural) distance-learning adult students. As well, we note that it is not possible to study multiple variants of SES as a determinant of metabolic outcome between the two cohorts. For example, Thai cohort members had homogenous levels of education, as they were recruited from Open University compared with the Pelotas cohort. We also note differences in SES measures: continuous income was reported in the Pelotas cohort but categorical income was reported in the Thai cohort with the middle-income group being the largest. Unfortunately it was not possible to create a smaller group. However, as an SES relative measure we do not envisage a major impact on the study conclusion.

Despite great differences in culture, ecology and medical geography of the two cohorts, we were able to explore the health-risk transition pathway for gender–SES effects. We confirmed that an increase in body size is already visible in early adulthood set within emerging economies. Evidently both Brazil and Thailand have the appropriate socio-cultural determinants that resulted in females switching to an inverse SES body weight pattern. Mechanisms for this phenomenon require further investigation. As both cohorts age, longitudinal data on early lifecourse exposure or outcomes such as SES and obesity will provide insight into how the gender–SES–weight pattern inverts or proceeds into later years.

## Figures and Tables

**Table 1:** Pelotas and Thai cohort characteristics by sex at baseline

*Cohort characteristics*^a^	*Pelotas 1982 Birth Cohort (*n=*5914)*			*Thai cohort 2005 (*n=*9893)*		
	*Total % (*n*)*	*Male*	*Female*	*Total % (*n*)*	*Male*	*Female*
*Socio-demographic characteristics*						
Male	51.3 (3037)			32.4 (3207)		
Live in urban area	100.0 (5914)			42.9 (4224)		
9-year school completion	63.8 (2739)	58.2	69.6	99.3 (9800)	98.9	95.5
Socioeconomic status (income)^b^						
SES 1 (lowest)	33.2 (1078)	31.1	36.5	20.2 (2003)	24.8	18.1
SES 2	33.5 (1089)	33.2	32.8	47.5 (4695)	39.1	51.5
SES 3 (highest)	33.3 (1080)	35.7	30.7	32.3 (3195)	36.1	30.5
						
*Body mass index baseline 2004/2005*^b^						
Underweight (BMI<18.5)	5.8 (187)	4.4	7.1	25.2 (2467)	13.0	31.0
Normal (BMI 18.5–<25)	64.9 (2106)	64.0	65.7	67.0 (6569)	76.0	62.7
Overweight (BMI 25–<30)	20.7 (673)	23.4	18.0	6.0 (596)	8.6	4.9
Obese BMI (30+)	8.6 (280)	8.1	9.1	1.7 (170)	2.4	1.4
						
*BMI baseline (Asian cutoff)*						
Overweight at risk (BMI 23–<25)				8.1 (798)	13.1	5.8
Overweight and obese BMI (25+)				7.8 (766)	10.9	6.3
						
*Body mass index follow-up 2012/2013*						
Underweight (BMI<18.5)	2.0 (71)	1.5	2.5	11.3 (1106)	5.1	14.2
Normal (BMI 18.5–<25)	40.5 (1438)	35.6	45.2	67.9 (6661)	66.3	68.7
Overweight (BMI 25–<30)	34.6 (1228)	40.7	18.6	15.8 (1548)	22.3	12.7
Obese BMI (30+)	22.9 (814)	22.1	23.7	5.1 (497)	6.3	4.5
						
*BMI follow up (Asian cutoff)*						
Overweight at risk (BMI 23–<25)				16.4 (1606)	22.9	13.2
Overweight and obese BMI (25+)				20.8 (2045)	28.6	17.1
						
*Health behaviors*						
Regular smoking	25.7 (2721)	27.6	23.6	5.5 (541)	15.3	0.6
Regular alcohol drinking	17.6 (757)	18.6	12.9	17.9 (1805)	37.8	8.0
Physical activity (>150 min per week)	68.8 (2956)	78.8	58.3	66.7 (6710)	81.0	66.5

Abbreviations: BMI, body mass index; SES, socioeconomic status.

a Born in 1982, followed up in 2004/2005; Thai cohort born between 1980 and 1985, followed up in 2005.

b Proportion was reported for cohort members that were successfully followed up in 2012.

**Table 2:** Pelotas and Thai cohorts: socioeconomic status and body mass index by sex at baseline and at 8-year follow up

*Cohorts*	*Body mass index (BMI)*^a^	*BMI column % (*n*) by socioeconomic status (SES) income groups and sex*							
		*Males*			*χ*^*2*^^b^ P*-value*	*Females*			*χ*^*2*^ P*-value*
		*SES 1*	*SES 2*	*SES 3*		*SES 1*	*SES 2*	*SES 3*	
Pelotas	2004^c^								
	Underweight	4.6 (22)	5.4 (38)	3.3 (19)	*P*=0.003	6.9 (41)	6.3 (34)	8.2 (41)	*P*<0.001
	Normal	69.9 (337)	60.9 (337)	62.0 (357)		59.9 (357)	64.6 (346)	73.9 (372)	
	Overweight	17.8 (86)	25.9 (143)	25.9 (149)		21.1 (126)	18.3 (98)	14.1 (71)	
	Obese	7.7 (37)	7.8 (43)	8.9 (51)		12.1 (72)	10.8 (58)	3.8 (19)	
									
	2012 follow up								
	Underweight	1.8 (9)	1.8 (10)	0.9 (5)	*P*=0.090	2.3 (14)	2.4 (13)	2.2 (11)	*P*<0.001
	Normal	39.0 (188)	34.2 (189)	33.3 (192)		39.9 (238)	41.4 (222)	53.7 (270)	
	Overweight	37.6 (181)	39.4 (218)	44.5 (257)		30.8 (184)	29.3 (157)	25.5 (127)	
	Obese	21.6 (104)	24.6 (136)	21.3 (123)		26.8 (160)	26.8 (144)	18.8 (95)	
									
Thai	2005								
	Underweight	16.6 (131)	12.8 (158)	10.7 (84)	*P*=0.333	32.2 (385)	30.8 (1051)	30.6 (619)	*P*=0.103
	Normal	75.2 (571)	77.0 (954)	77.3 (888)		60.2 (719)	62.9 (2147)	63.8 (1290)	
	Overweight	8.1 (64)	7.7 (95)	9.9 (114)		5.8 (69)	5.0 (172)	4.0 (82)	
	Obese	2.7 (21)	2.5 (31)	2.0 (23)		1.7 (21)	1.5 (44)	1.3 (30)	
									
(Asian cutoffs)	Overweight (23–<25)	10.7 (84)	13.4 (166)	14.5 (166)	*P*=0.207	6.1 (73)	6.1 (209)	4.9 (100)	*P*=0.050
	Obese (25+)	10.8 (85)	10.2 (126)	11.9 (137)		7.5 (90)	6.3 (216)	5.5 (112)	
									
	2013 follow up								
	Underweight	6.7 (53)	4.9 (61)	4.3 (49)	*P*=0.010	16.0 (191)	13.8 (470)	13.9 (282)	*P*=0.059
	Normal	65.9 (516)	68.2 (846)	63.8 (833)		67.3 (804)	68.3 (2333)	70.1 (1417)	
	Overweight	20.6 (162)	20.3 (252)	25.7 (295)		13.7 (136)	11.4 (468)	11.6 (235)	
	Obese	6.8 (57)	6.6 (82)	6.2 (60)		5.4 (64)	4.5 (147)	4.3 (87)	
									
(Asian cutoffs)	Overweight (23–<25)	20.6 (162)	24.4 (303)	22.9 (263)	*P*=0.012	14.6 (174)	12.9 (440)	13.0 (264)	*P*=0.049
	Obese (25+)	27.8 (219)	26.9 (334)	30.9 (355)		16.7 (200)	18.0 (615)	15.9 (322)	
									

a Western standard body mass index: <18.5 as ‘underweight', 18.5–<25 ‘normal', 25–<30 ‘overweight' and 30+ ‘obese'.

b *χ*^2^ *P*-values represent statistically significant differences between ‘normal' vs ‘overweight/obese' body mass index across SES groups.

c Proportion was reported for cohort members that were successfully followed up in 2012.
